# Genomic epidemiology of carbapenem-resistant Enterobacterales at a New York City hospital over a 10-year period reveals complex plasmid-clone dynamics and evidence for frequent horizontal transfer of *bla*_KPC_

**DOI:** 10.1101/gr.279355.124

**Published:** 2024-11

**Authors:** Angela Gomez-Simmonds, Medini K. Annavajhala, Dwayne Seeram, Todd W. Hokunson, Heekuk Park, Anne-Catrin Uhlemann

**Affiliations:** Division of Infectious Diseases, Department of Medicine, Columbia University Irving Medical Center, New York, New York 10032, USA

## Abstract

Transmission of carbapenem-resistant Enterobacterales (CRE) in hospitals has been shown to occur through complex, multifarious networks driven by both clonal spread and horizontal transfer mediated by plasmids and other mobile genetic elements. We performed nanopore long-read sequencing on CRE isolates from a large urban hospital system to determine the overall contribution of plasmids to CRE transmission and identify specific plasmids implicated in the spread of *bla*_KPC_ (the *Klebsiella pneumoniae* carbapenemase [KPC] gene). Six hundred and five CRE isolates collected between 2009 and 2018 first underwent Illumina sequencing for genome-wide genotyping; 435 *bla*_KPC_-positive isolates were then successfully nanopore sequenced to generate hybrid assemblies including circularized *bla*_KPC_-harboring plasmids. Phylogenetic analysis and Mash clustering were used to define putative clonal and plasmid transmission clusters, respectively. Overall, CRE isolates belonged to 96 multilocus sequence types (STs) encoding *bla*_KPC_ on 447 plasmids which formed 54 plasmid clusters. We found evidence for clonal transmission in 66% of CRE isolates, over half of which belonged to four clades comprising *K. pneumoniae* ST258. Plasmid-mediated acquisition of *bla*_KPC_ occurred in 23%–27% of isolates. While most plasmid clusters were small, several plasmids were identified in multiple different species and STs, including a highly promiscuous IncN plasmid and an IncF plasmid putatively spreading *bla*_KPC_ from ST258 to other clones. Overall, this points to both the continued dominance of *K. pneumoniae* ST258 and the dissemination of *bla*_KPC_ across clones and species by diverse plasmid backbones. These findings support integrating long-read sequencing into genomic surveillance approaches to detect the hitherto silent spread of carbapenem resistance driven by mobile plasmids.

The prevalence of carbapenem-resistant Enterobacterales (CRE) in the United States and worldwide has increased substantially in recent decades, driven primarily by the rapid dissemination of carbapenem-hydrolyzing β-lactamases (Castanheira et al. 2019). Most clinically important carbapenemases, such as the *Klebsiella pneumoniae* carbapenemase (KPC), are typically encoded by plasmids (Mathers et al. 2015). In the United States, where the dominant mechanism of carbapenem resistance in Enterobacterales is KPC production, the molecular epidemiology of CRE has been shown to be complex, consisting both of highly successful clones such as *K. pneumoniae* sequence type (ST) 258 and diverse lineages that have acquired *bla*_KPC_ (the β-lactamase gene encoding KPC) by plasmid uptake (Cerqueira et al. 2017; Lapp et al. 2023). The presence of multiple co-occurring mechanisms for *bla*_KPC_ dissemination, together with the potential for horizontal transfer of *bla*_KPC_-harboring plasmids to increase CRE diversity, underscore the challenges of investigating CRE transmission dynamics and implementing effective surveillance approaches.

Prior studies have implicated a wide variety of plasmids contributing to the spread of *bla*_KPC_, both through their association with successful clones and transmissibility among unrelated bacteria (Mathers et al. 2015; Navon-Venezia et al. 2017). The plasmids most frequently encoding *bla*_KPC_ belong to the relatively narrow host range IncF family. This includes IncFIB(pQkil) and IncFIB/FII(K)-type plasmids, which are conserved within ST258 lineages and likely played an important role in the global spread of CRE (Chen et al. 2014b; Bowers et al. 2015). In contrast, broad host range plasmids, such as IncN-type plasmids, have been shown to contribute to the transmission of *bla*_KPC_ between different bacterial strains and species (Conlan et al. 2014; Gomez-Simmonds et al. 2022). In many plasmids, *bla*_KPC_ is found within *Tn*4401 transposons (Kitchel et al. 2009; Chen et al. 2014c), often integrated within other transposons (Sheppard et al. 2016), which may further enable its transmission to new plasmid backbones and bacterial hosts.

Illumina sequencing is the most widely used sequencing platform for genomic surveillance of CRE and other multidrug-resistant pathogens but can be challenging to use for plasmid investigation. Plasmid genetic heterogeneity and plasticity limit the reliability of plasmid typing schemes and reference-based approaches and can encumber de novo assembly from short-read sequencing data (Sheppard et al. 2016; Orlek et al. 2017). Long reads are able to resolve complex and repetitive genetic regions found on plasmids to enable detailed analysis of plasmid structures (George et al. 2017). Long-read sequencing platforms (e.g., PacBio, Oxford Nanopore Technologies [ONT]) have become increasingly accessible in recent years, during which time multiple studies have been published highlighting the role of plasmids in the transmission of carbapenem resistance genes (Conlan et al. 2014; Sheppard et al. 2016; David et al. 2020; Shropshire et al. 2022; Lerminiaux et al. 2023; Roberts et al. 2023). While these studies broadly advocate for expanding genomic surveillance to account for horizontal transfer, its potential impact remains unclear both because the proportion of CRE attributable to plasmid-mediated transmission of carbapenemase genes and specific targets for surveillance have yet to be clearly delineated. Here, we characterized the population structure of *bla*_KPC_-harboring plasmids at a large New York City medical center and determined the contribution of plasmids versus bacterial clones to the spread of *bla*_KPC_ over a 10-year period. We performed both short- and long-read sequencing and hybrid assembly on all isolates included in the analysis to provide a complete assessment of plasmid diversity and find evidence for horizontal transfer. Our goals were to estimate the incidence of plasmid-mediated transmission of *bla*_KPC_ and identify promiscuous plasmid lineages that should be included in future surveillance efforts.

## Results

### Clinical isolate characteristics

**Figure 1.**
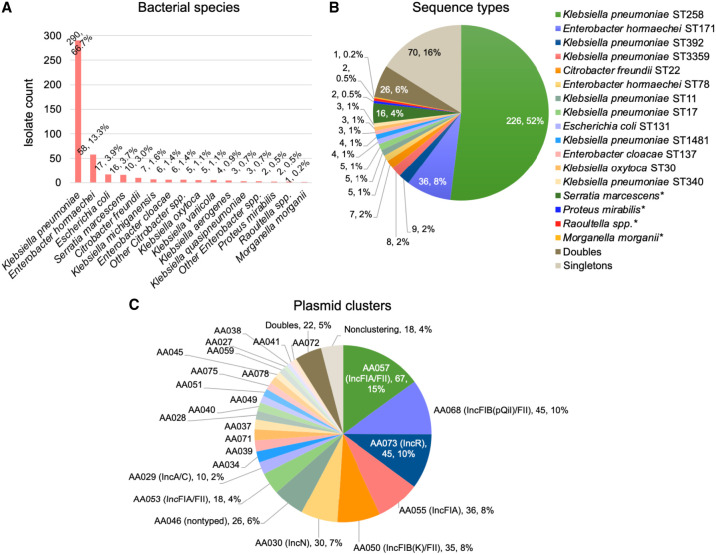
Population structure of CRE isolates. Number and proportion of CRE isolates harboring *bla*_KPC_ (*n* = 460) that belonged to each (*A*) bacterial species and (*B*) ST. Doubles refer to STs that include two isolates; singleton STs consist of only one isolate. (*C*) Diversity of plasmids harboring *bla*_KPC_. As above, the term doubles refers to plasmid clusters that include two plasmids. The small proportion of plasmids that did not share a secondary MOB-cluster designation with any other plasmid are denoted as nonclustering.

We identified 575 patients with a clinical culture positive for CRE during the study period (2009–2018) that were part of a large retrospective collection of multidrug-resistant isolates collected at our medical center (Gomez-Simmonds et al. 2018a,b; Macesic et al. 2020; Gomez-Simmonds et al. 2022) and had an isolate(s) available for genomic analysis. A total of 605 isolates representing a unique bacterial species from each patient were included in the study. These isolates were collected from a variety of body sites, including bloodstream (18%), respiratory (29%), intra-abdominal (5%), urinary tract (33%), and wound cultures (6%). Most isolates were cultured from intensive care unit (ICU) patients (39%) or non-ICU inpatients (43%) although a subset were obtained in the emergency department (10%) or outpatient setting (8%). *bla*_KPC_ was identified in 460/605 (76%) isolates (39% *bla*_KPC-2_; 58% *bla*_KPC-3_; 2% *bla*_KPC-4_; 0.5% *bla*_KPC-56_) demonstrating substantial species and clonal diversity (Fig. 1A,B). Of these, we were able to generate complete hybrid assemblies for 435/460 (95%) isolates encoding *bla*_KPC_ (Supplemental Table S1), which included single-contig, circular assemblies for all *bla*_KPC_-harboring plasmids. Overall *bla*_KPC_-harboring CRE isolates consisted of 22 unique bacterial species, the most frequently identified of which were *K. pneumoniae* (67%), *Enterobacter hormaechei* (13%), and *Escherichia coli* (4%). Of 96 different clones defined by multilocus sequence type (MLST; six bacterial species represented in this collection lack an MLST scheme), *K. pneumoniae* ST258 was by far the most common, comprising over half of isolates (52%), followed by *Enterobacter cloacae* ST171 (8%) and *K. pneumoniae* ST392 (2%). All other clones comprised ≤8 isolates, including 70 (16%) singletons.

For comparison, we generated Illumina short-read assemblies for six isolates in which *bla*_KPC_ was found on six different plasmid types (see Supplemental Fig. S1). All short-read-assembled plasmids were subdivided into 12–62 contigs, and the assembler was unable to resolve the location of *bla*_KPC_ and its associated *Tn*4401 transposon for 3/6 isolates, consistent with low plasmid contiguity for the Illumina assemblies.

### Genetic context of *bla*_KPC_ in clinical isolates

Long-read sequencing and hybrid assembly of 435 isolates in our collection enabled detailed analysis of the genomic location of *bla*_KPC_. *bla*_KPC_ was found on the bacterial chromosome in 14 isolates (3%), on the chromosome and at least one plasmid in 17 isolates (4%), on a single plasmid in 381 isolates (88%), and on ≥2 plasmids in 23 isolates (5%). Overall, *bla*_KPC_ was found on 447 plasmids. These plasmids included many different plasmid replicon types, most often belonging to the IncF family as well as IncN, IncA/C, IncR, IncL/M, IncI2, ColRNAI, and nontypeable plasmids using this scheme. Multireplicon plasmids were also common (201/447, 45%), usually involving the presence of two or more IncF replicons.

In this collection, *bla*_KPC_ was encoded within a variant of the *Tn*4401 transposon in all isolates but one (37% *Tn*4401a; 28% *Tn*4401b; 34% *Tn*4401d; 2% *Tn*4401e). Small deletions within *Tn*4401b involving *tnp*A and *tnp*R were seen in 10 isolates, while larger structural changes in *Tn*4401 occurred in three isolates. This included two isolates with truncated *Tn*4401Δa (pKP0100 and pNR5650) and one isolate with insertion of a 9760 bp fragment encoding *IS*Kpn19 and *IS*Ror7 associated with the formaldehyde resistance genes *frmA*, *frmB*, and *frmR* located between *tnpA* and *IS*Kpn7 in *Tn*4401Δb (pNR2980). In one isolate, *bla*_KPC-2_ was associated with an NTE4-like element (pNR2252) (Bonnin et al. 2020). Fifty-one (12%) isolates had ≥2 copies of *bla*_KPC_. All isolates with multiple copies of *bla*_KPC_ were found to have both the same *bla*_KPC_ allele and *Tn*4401 isoform, and may have represented transposition of *Tn*4401 to a new plasmid or chromosomal site rather than new plasmid uptake.

### Plasmid clustering analysis

We next classified *bla*_KPC_-harboring plasmids using MOB-cluster with the goal of identifying groups of plasmids likely to belong to the same transmission network. MOB-cluster applies two different mash distance thresholds to generate clusters consisting of similar (primary MOB-cluster; mash distance threshold of 0.06) and near-duplicate (secondary MOB-cluster; mash distance threshold of 0.025) plasmids (Robertson et al. 2023). Here, we found that the 447 plasmids in this collection belonged to 26 primary and 54 secondary MOB-clusters, respectively. Only 3 (0.6%) plasmids did not share a primary cluster designation with at least one other plasmid and were considered sporadic; 18 (4%) plasmids were the only members of their secondary plasmid cluster (Fig. 1C). For the purposes of this analysis, we chose to define plasmid clusters using their more stringent secondary MOB-cluster designations; thus, in the analyses described below, “plasmid clusters” refers to secondary MOB-clusters unless otherwise specified.

**Figure 2.**
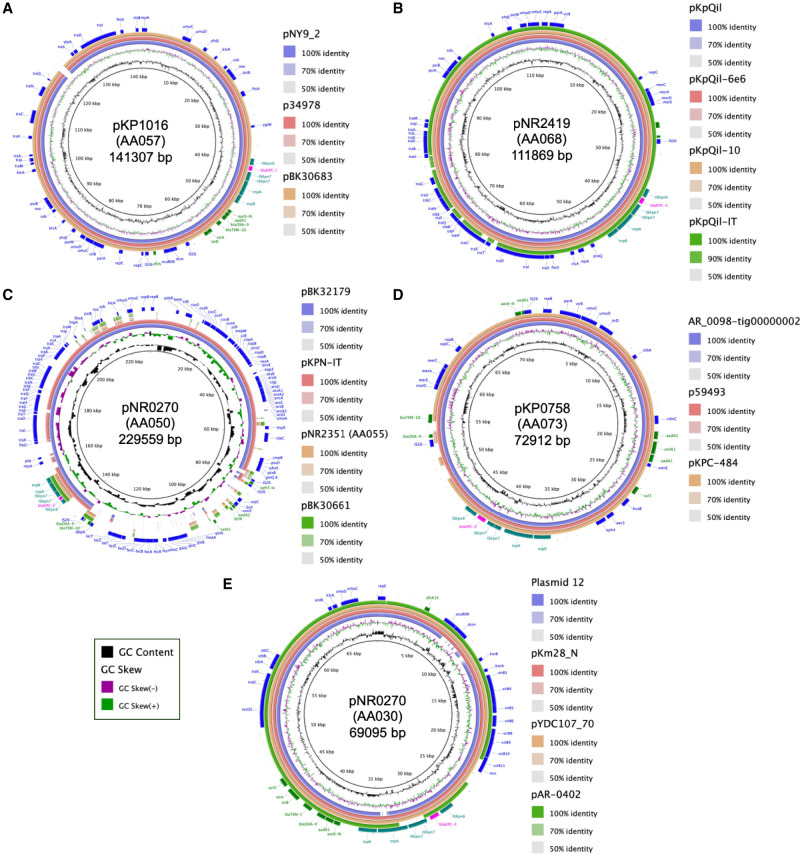
Comparison of most frequently identified plasmids from this study with publicly available plasmids including those with widespread distribution. Alignment of representative plasmids from each of the largest plasmid clusters against similar, publicly available plasmid genomes. (*A*) AA057; (*B*) AA068; (*C*) AA050 and AA057; (*D*) AA073; and (*E*) AA030. For all plasmid annotations, *bla*_KPC_ is shown in pink and Tn4401-associated genes in teal, other antibiotic resistance genes in green, and other genes in blue.

The median cluster size was six plasmids (interquartile range [IQR] 2–9.5) and only nine clusters comprised ≥10 plasmids (Fig. 1C). Five of the largest plasmid clusters belonged to the IncF plasmid family (representative plasmids for each large plasmid cluster are shown in Supplemental Fig. S1). The largest cluster, AA057 (*n* = 67 plasmids, 15%), consisted of IncFIA/FII plasmids found in *K. pneumoniae* ST258 and *E. cloacae* ST171 as well as in other diverse strains as described in more detail below. AA057 plasmids were highly similar to IncFIA plasmids previously identified in carbapenem-resistant *E. hormaechei* and *K. pneumoniae*, including pBK30683 (NC_025131), which was found to be widely distributed in hospitals in New York and New Jersey (Fig. 2A; Chen et al. 2014a; Chavda et al. 2016; Huang et al. 2017; Gomez-Simmonds et al. 2018a). Alignment of representative AA057 plasmid pKP1016 to PLSDB, a resource for accessing plasmid genomes from the NCBI nucleotide database, revealed the presence of closely related plasmids in many different Enterobacterales species distributed across the United States, China, and Europe (Galata et al. 2019). Two other large IncF/FII plasmid clusters, AA068 (*n* = 45, 10%) and AA050 (*n* = 35, 8%), consisted of plasmids with strong sequence homology with the widespread pKpQil (GU595196) and the pKPN3-like plasmids (e.g., pBK32179; CP020838), respectively (Fig. 2B,C; Leavitt et al. 2010; Chen et al. 2013). While pKpQil and *bla*_KPC_-harboring pKPN3-like plasmids are also globally distributed, they have been primarily associated with *K. pneumoniae* and in our collection were almost all identified in ST258 isolates. Similarly, the IncFIA plasmid cluster AA055 (*n* = 36, 8%) was identified mostly in ST171 isolates. AA055 plasmids closely resembled pBK30661 (KF954759) (Fig. 2C), which was previously shown to have a 72 kb sequence that is highly similar to pBK30683, but lacks an origin of transfer (*oriT*) locus and *tra* operon required for conjugation (Chen et al. 2014a).

The largest non-IncF-type plasmid cluster (AA073, *n* = 45, 10%) consisted of IncR plasmids that harbored multiple antibiotic resistance genes and were predicted to be nonconjugative. These plasmids were almost all found in ST258 as well as the ST258 single-locus variant ST1481. Most similar plasmids identified in PLSDB were also found in *K. pneumoniae*, and further investigation of top hits including pKPC-484 (CP008798) and p59493 (CP068609) revealed that they were also derived from ST258 isolates (Fig. 2D; Conlan et al. 2014; Hao et al. 2021). In contrast, the conjugative IncN plasmids making up cluster AA030 (*n* = 30, 7%) were found in 24 different STs. In a previous study, we found that these IncN plasmids were both highly transmissible and persistent based on their close identity with the first fully sequenced *bla*_KPC-3_-harboring plasmid at our hospital from 2005, plasmid 12 (FJ223605) (Fig. 2E; Gootz et al. 2009; Gomez-Simmonds et al. 2022). Top hits in PLSDB included plasmids found in *E. coli* (pYDC107_70; CP025710), *Klebsiella oxytoca* and *Klebsiella michiganensis* plasmids collected at a New York City hospital in 1997 (pKm38_N; KY128483), and a *bla*_KPC_-encoding plasmid in *Salmonella enterica* (pAR-0402; CP044187), further corroborating that these plasmids have been circulating for many years in highly diverse hosts (Eilertson et al. 2017; Hazen et al. 2018). Smaller plasmid clusters in this collection (<30 plasmids) belonged to the IncF, IncN, IncC, IncR, or other plasmid families, and several were nontypeable.

Analysis of the longitudinal distribution of these plasmid types over time revealed that despite changes in overall number of CRE isolates and STs, the distribution of plasmids remained consistent throughout the study period (Supplemental Fig. S2). Among the 36 plasmid clusters, 13 were found to persist for at least 5 years of the study period, including all nine plasmid clusters that included at least 10 plasmids. Furthermore, among the most prevalent plasmid clusters, the relative proportion of plasmids also did not appear to change substantially over time.

### CRE clones and associated plasmids

We performed a phylogenetic analysis of CRE genomes to identify closely related isolates within each ST and examine the distribution of plasmids within and across clonal sublineages. There were 364 isolates sharing an ST (or species for organisms lacking an MLST scheme) with at least one other isolate. Among these, we identified 288 (66% of 435 isolates with complete sequencing data) isolates meeting the criteria for inclusion in one of 18 clades defined by grouping isolates from the same ST with pairwise genetic distances of ≤25 single-nucleotide polymorphisms (SNPs) (see Supplemental Fig. S3). *K. pneumoniae* ST258 (four clades) and *E. cloacae* ST171 (two clades) were the only STs with ≥2 clades of closely related isolates after applying this criterion and the only STs that had a clade consisting of >7 isolates. All other STs comprised only one small clade of closely related isolates, often with additional branches representing more distantly related isolates (i.e., isolates that differed by >25 SNPs), indicating that outside of ST258 and ST171 clonal transmission was relatively limited.

**Figure 3.**
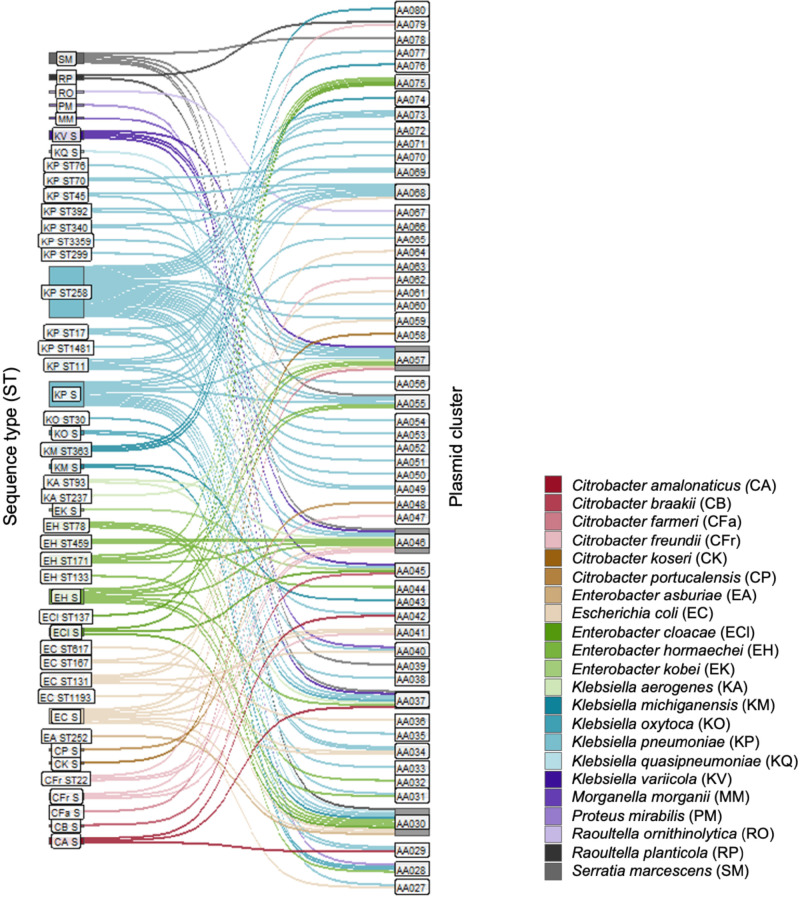
Plasmid distribution across different CRE STs. This Sankey plot demonstrates the relationship between isolated STs and plasmid clusters found in this collection; bacterial species are designated by color, demonstrating the host range for each plasmid cluster. STs consisting of only one isolate are labeled as singletons (S). Bar sizes correspond to the number of plasmids in each ST and plasmid cluster.

**Figure 4.**
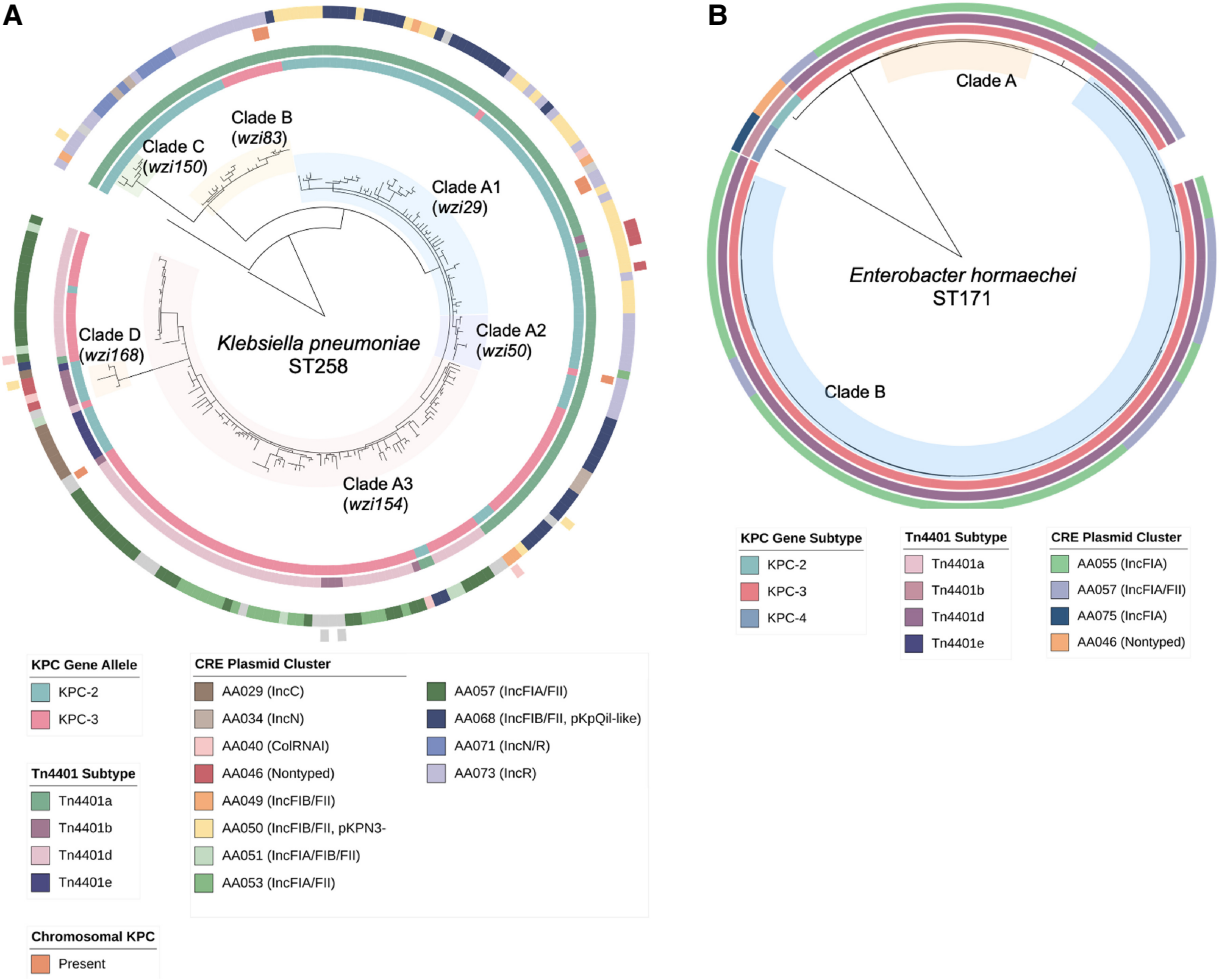
Clade and plasmid distribution in *K. pneumoniae* ST258 and *E. hormaechei* ST171 isolates. For (*A*) ST258 and (*B*) ST171 phylogenetic trees, the annotations denote the *bla*_KPC_ allele (inner ring) and associated *Tn*4401 subtype (second ring) as well as the genetic location of *bla*_KPC_ (chromosome and plasmid cluster; outer rings) for each isolate. Clade designations refer to groups of isolates within each ST with pairwise genetic distances of ≤25 SNPs.

When we examined the distribution of plasmids across different CRE clones (Fig. 3), we found that most STs were associated with a small number of plasmid clusters. Similarly, 10/18 clades identified in our phylogenetic analysis had *bla*_KPC_-encoding plasmids belonging to a single plasmid cluster. ST258, which harbored plasmids belonging to 18 different plasmid clusters, was the only clone associated with >5 plasmid clusters. Among the four ST258 clades, the largest (clade A) was associated with plasmids from 16 different plasmid clusters, and the other three clades harbored three plasmid clusters (Fig. 4A). In contrast, most ST171 isolates belonged to one large clade (clade B), primarily consisting of isolates with *bla*_KPC_ encoded on an AA055 plasmid (Fig. 4B), and some isolates harboring the similar AA057 plasmids. AA055 plasmids were also found in all ST171 isolates belonging to a second, smaller clade.

**Figure 5.**
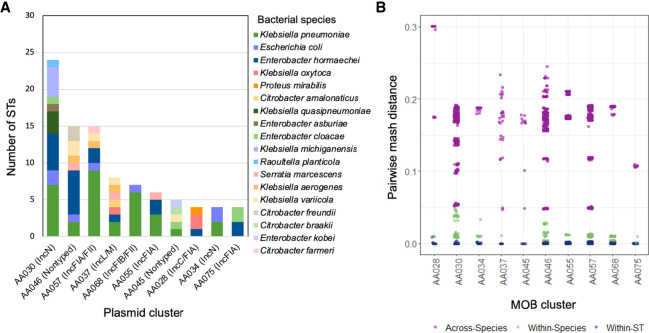
Top 10 plasmids demonstrating the greatest host diversity. (*A*) Plasmid clusters found in the greatest number of isolate STs. (*B*) Analysis of pairwise mash distances between plasmid host genomes corresponding to within-strain and species and across species genetic differences demonstrated the potential for distribution of these plasmids to diverse hosts.

We then further characterized plasmids that were found in the greatest number of unrelated bacterial isolates (Fig. 5A). Overall, these “promiscuous” plasmid clusters belonged to several different plasmid families, including IncA/C, IncN, IncL/M, IncF, IncR, ColRNAI, and nontypeable plasmids. The IncN plasmid AA030 was identified in the greatest number of unique clones in this collection, as it was detected in 30 isolates belonging to 24 different STs from eight bacterial species. The IncFIA/FII plasmid AA057 was found in closely related ST258 and ST171 isolates as well as in an additional 13 STs, suggesting that it has genetic factors enhancing both stability and transmissibility. The small, nontypeable plasmid AA046 was distributed among 14 different STs. All plasmids belonging to this cluster were predicted to be mobilizable but nonconjugative despite being found in diverse hosts, indicating that non-self-transferrable plasmids may still be capable of dissemination. This plasmid was also present in five isolates coharboring the pKPN-like plasmid AA050, which is known to have a narrow host range and was restricted to ST258 in this collection, and thus may facilitate transmission of *bla*_KPC_ from these isolates. Finally, we also identified a broad host range of IncL/M plasmid (AA037) in eight different STs; previously, IncL/M plasmids have rarely been implicated in the transmission of *bla*_KPC_ (Rozwandowicz et al. 2018). In an assessment of pairwise Mash distances between bacterial host genomes (Fig. 5B), we found that plasmids belonging to these plasmid clusters were distributed across diverse isolates within and across different bacterial species.

### Plasmid variability within ST258

Given the considerable plasmid diversity found in ST258 isolates, we examined this clone more closely to assess the distribution of specific plasmids across the ST258 phylogenetic tree (see Fig. 4A). The largest clonal sublineage (clade A, *n* = 189) harbored *bla*_KPC_-encoding plasmids belonging to several different plasmid clusters predominantly from the IncF family, including AA050 (pKPN3-like, *n* = 33), AA068 (pKpQil-like, *n* = 37), and AA057 (IncFIA/FII, *n* = 42). However, IncN, IncR, IncC, ColRNAI, and other plasmid families were also represented within this cluster. When we subdivided clade A into three subclades based on *wzi* typing (clades A1–A3), we found that each subclade was associated with specific plasmid clusters; for example, the predominant plasmid clusters in clades A1 and A3 were AA050 and AA057, respectively, although AA068 plasmids were also interspersed throughout both subclades. Clade B consisted of 24 isolates mostly harboring IncR (AA073) and IncN/R (AA071) plasmids, which belonged to the same primary MOB-cluster, whereas clades C and D were smaller and had several different *bla*_KPC_-harboring plasmids including sporadic plasmid types.

**Figure 6.**
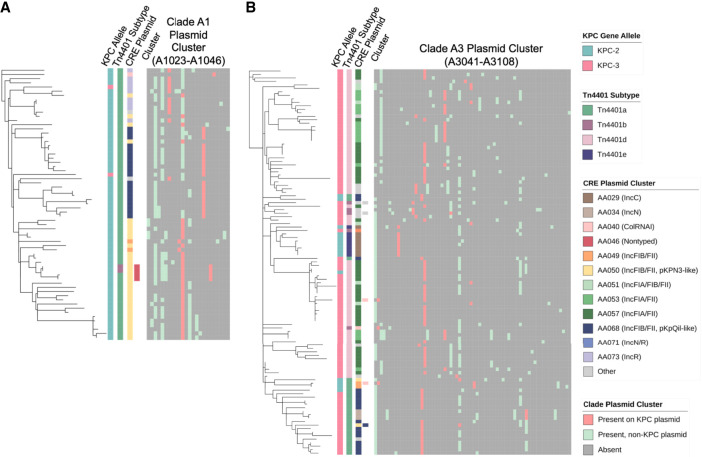
Distribution of *bla*_KPC_-harboring plasmids and non-*bla*_KPC_-harboring plasmids across ST258 clades. Phylogenetic trees for isolates belonging to (*A*) clade A1 and (*B*) clade A3, with corresponding distribution of *bla*_KPC_, *Tn*4401 subtype, and CRE plasmid cluster as shown previously, as well as results of MOB-clustering analysis including both *bla*_KPC_-harboring and non-*bla*_KPC_-harboring plasmids.

To further explore this dynamic process, we examined the genetic context of *bla*_KPC-2_ as well as the interplay between *bla*_KPC_-harboring and all other plasmids found in clades A1 and A3 isolates. Almost all clade A1 isolates (63/65) had *bla*_KPC-2_ associated with a *Tn*4401a transposon (Fig. 6A). Plasmid clustering analysis including *bla*_KPC_ and non-*bla*_KPC_-harboring plasmids revealed that most isolates in clade A1 harbored AA050 plasmids, many of which harbored *bla*_KPC_, although in two subclades *bla*_KPC_ was found predominantly on AA068 and AA073 plasmids. This may suggest that the expansion of this clade followed the acquisition of the AA050 plasmid, and was followed by the mobilization of *Tn*4401a to new plasmid backbones. Within clade A3 (Fig. 6B), we identified several branches with distinct *Tn*4401 subtypes and plasmid clusters, potentially suggestive of new *bla*_KPC_-harboring plasmid acquisition and/or exchange followed by clonal spread in these branches. Here *bla*_KPC_-harboring and non-*bla*_KPC_-harboring plasmids appeared to be distributed across distinct clusters, unlike what was seen in clade A1 isolates. Overall, this suggests that both transposition and plasmid uptake contributed to plasmid diversity within ST258 clades.

### Contribution of plasmid transmission to hospital CRE

Based on the identification of clusters of closely related isolates and plasmids as described above, we then aimed to determine what proportion of CRE isolates could be attributed to clonal transmission versus acquisition of a *bla*_KPC_-harboring plasmid by horizontal transfer (see Supplemental Fig. S3). In our complete collection of 435 isolates, we again found that 288 (66%) isolates met the criteria for presumed clonal transmission. Of the remaining 147 isolates, 127 (29%) had a *bla*_KPC_-harboring plasmid belonging to a plasmid cluster. Thirty-nine (9%) of these isolates had *bla*_KPC_ encoded by the same plasmid as at least one other isolate from the same ST and thus may also have belonged to the same clonal transmission network but did not meet the criteria for clonal transfer. However, 88 (20%) isolates shared a plasmid from the same plasmid cluster only with unrelated isolates, suggesting plasmid-mediated acquisition of *bla*_KPC_ in these isolates. In 20 (5%) isolates sharing neither the same ST nor plasmid with another isolate, the mechanism of *bla*_KPC_ transmission could not be determined. Thus, the incidence proportion of plasmid transmission in the full isolate collection was estimated to be 23%.

When we limited this analysis to the 303 isolates collected between 2014 and 2018, for which more comprehensive sample collection was available, we found that 187 (62%) isolates met the criteria for clonal transmission. Of the remaining 116 isolates, 98 (32%) shared a plasmid from the same cluster with at least one other isolate; 30 (10%) of these belonged to the same ST. The remaining 68 (22%) unrelated isolates had plasmids belonging to a plasmid cluster again suggestive of horizontal transfer, giving an incidence proportion of plasmid transmission of 27%.

The above analysis was based on isolates collected from clinical cultures and thus did not account for the potential for subclinical CRE to contribute to the spread *bla*_KPC_ by horizontal transfer. To determine whether active surveillance could improve the detection of putative plasmid transmission, we analyzed CRE isolates cultured from fecal samples from a prospective cohort of liver transplant recipients at our hospital undergoing active longitudinal surveillance for multidrug-resistant bacteria. In a previous description of this cohort, colonizing CRE isolates were found to form putative transmission networks with isolates collected from other hospitalized patients, suggesting that these patients may be reservoirs for CRE transmission within the hospital (Macesic et al. 2018). From this cohort, we identified 10 patients who had a *bla*_KPC_-positive CRE isolate cultured from a fecal sample between 2014 and 2018. Eleven different *bla*_KPC_-harboring plasmids from these patients were included in a clustering analysis with all 447 plasmids from our CRE isolate collection (Supplemental Table S2). Here 10/11 plasmids belonged to the same plasmid cluster (again defined by secondary MOB-cluster designation) as a plasmid from a clinical CRE isolate. Furthermore, the active surveillance plasmids almost all belonged to a large plasmid cluster (9/11 plasmids belonged to a plasmid cluster with >10 plasmids), including AA057, AA068, AA073, AA050, and AA030. Thus active surveillance in this high-risk patient population did not lead to the identification of a substantial number of additional putative plasmid transmission events.

## Discussion

Based on performing comprehensive short- and long-read sequencing and hybrid assembly on a large data set of clinical CRE isolates, we found evidence for both clonal dominance by *K. pneumoniae* ST258 and dissemination of *bla*_KPC_ across clones and species driven by diverse plasmids. We also estimated the incidence of plasmid-mediated *bla*_KPC_ transmission occurring among unrelated Enterobacterales to be ∼23%–27%. Finally, we identified several high-risk plasmids that appeared to play a prominent role in the spread of *bla*_KPC_ among unrelated bacterial clones and species. Our findings extend those of prior studies demonstrating that plasmids are important contributors to the diversification and spread of CRE despite substantial differences in CRE epidemiology between centers (Conlan et al. 2014; David et al. 2020; Shropshire et al. 2022; Yong et al. 2022; Lerminiaux et al. 2023; Roberts et al. 2023; Macesic et al. 2024). They also suggest the presence of several prominent plasmids that could be important targets for future surveillance efforts as they appeared to be highly transmissible and were linked to international, circulating plasmid lineages. Taken together, these findings strongly support the use of long-read sequencing in genomic surveillance approaches to previously unrecognized *bla*_KPC_ transmission driven by mobile plasmids.

The majority of CRE isolates in this collection belonged to the globally dominant clone ST258, which as in other studies was subdivided into several smaller sublineages consisting of closely related isolates (Chen et al. 2014c; DeLeo et al. 2014). *bla*_KPC_ was primarily found on IncF-type plasmids within ST258 isolates, consistent with the established relationship between this clone-plasmid pair, which has been thought to contribute epidemiologically to the success of this clone (Mathers et al. 2015; Peirano et al. 2017; Stoesser et al. 2017). Some plasmid clusters associated with ST258 and ST171 were also found in diverse, unrelated bacteria, such as the IncFIA/FII plasmid AA057. It is noteworthy that AA057 was the largest plasmid cluster in this collection and outnumbered pKpQil and pKPN3-like plasmids, both of which were largely restricted to ST258. While all three plasmid backbones could be linked to plasmids with global distribution, AA057 appears to have acquired a more expansive host range, possibly as a result of the acquisition of a second *tra* gene locus as previously described (Chen et al. 2014a). Plasmids with the ability to both stably reside in high-risk strains and spread to unrelated strains may be particularly problematic in hospital settings and in need of targeted surveillance. Finally, the identification of a variety of other plasmids within ST258 clades suggested a highly dynamic process involving plasmid acquisition and recombination in this clone.

We identified the IncN plasmid AA030 as the most promiscuous plasmid in this study, with evidence of horizontal transfer of *bla*_KPC_ into 19 isolates spanning the entire study period, indicating ongoing circulation of this plasmid. Transmissibility of the IncN plasmid AA030 may be governed by a range of factors, including both high-efficiency replication and conjugation systems and the ability to avoid restriction–modification and other bacterial defense systems (Jain and Srivastava 2013; Gomez-Simmonds et al. 2022; Shaw et al. 2023). However, here acquisition of the IncN plasmid did not appear to be accompanied by clonal transmission, which may suggest that despite high transmission efficiency this plasmid was not stably maintained by recipient strains. We also identified plasmids that appeared to be highly promiscuous but lacked conjugation machinery, suggesting that host-independent conjugation is not a requirement for the dissemination of these isolates. Additional studies are needed to better delineate specific plasmid factors contributing to the spread of these plasmids.

Plasmid-mediated CRE transmission is challenging to detect using traditional surveillance approaches and may also be less responsive to infection control interventions than those driven by clonal spread. In a study of CRE transmission conducted in a public hospital in Singapore by Marimuthu et al. (2022), reductions in clonal spread during the last quarter of the study period were attributed to the implementation of various infection control measures; however, putative plasmid-mediated transmission continued to increase during this period and ultimately exceeded clonal spread. Similarly, according to antibiogram data from our New York City hospital, peak CRE incidence occurred in 2010–2011 and was dominated by *K. pneumoniae.* The subsequent rapid decline in CRE was most likely due to the implementation of aggressive infection control measures, but was also characterized by increased diversity of CRE species. Taken together, this indicates a critical need to improve surveillance efforts to account for plasmid-mediated transmission of carbapenem resistance, and suggests a role for integrating long-read sequencing into genomic surveillance workflows to facilitate plasmid detection.

Our study had several limitations. This was a single-center, retrospective analysis. Access to bacterial isolates was based on prior archiving of culture samples and was limited before 2014 as described. We also only included a single isolate from each bacterial species for each patient, further limiting potential plasmid diversity. Approaches for evaluating plasmid relatedness have also not been widely studied or standardized, particularly in large collections of diverse plasmid types, likely related to the difficulty of obtaining complete plasmid sequencing data. Based on our prior observation of large-scale recombination events occurring in closely related plasmids, we selected an alignment-free, *k*-mer-based clustering approach to identify plasmid clusters (Robertson and Nash 2018). Finally, our main analysis was restricted to isolates obtained from clinical culture and therefore largely representative of infection episodes. However, in a plasmid clustering analysis including plasmids derived from a longitudinal cohort of liver transplant recipients undergoing prospective surveillance for multidrug-resistant bacteria, most *bla*_KPC_-harboring plasmids clustered with plasmids derived from clinical cultures. Although further studies based on active surveillance are needed to provide better estimates of the extent of plasmid-mediated transmission of CRE, this suggests that clinical cultures, which are more readily accessible for genomic surveillance, may provide an adequate representation of plasmid populations in hospitalized patients.

Taken together, although *K. pneumoniae* ST258 and other dominant CRE clones represented a large proportion of isolates over a 10-year study period, we identified at least three plasmid categories of epidemiologic interest. The first is widespread, largely IncF-type plasmids that have disseminated widely through epidemic clones such as *K. pneumoniae* ST258 and *E. cloacae* ST171 and showed evidence of spreading to other strains. The second is highly successful, broad host range plasmids such as IncN plasmids that may represent important targets for ongoing surveillance given their potential to drive the multispecies spread of *bla*_KPC_. Finally, we identified a large collection of heterogeneous plasmids that most likely acquired *bla*_KPC_ sporadically by MGE transmission and are difficult to track. The relatively large proportion of CRE episodes attributable to plasmid-mediated transmission suggests an important role for the ongoing surveillance of *bla*_KPC_-harboring plasmids. This work also highlights the substantial diversity of CRE in an endemic setting and the utility of using comprehensive plasmid sequencing for elucidating transmission networks.

## Methods

### Isolate selection

We identified and collected all available CRE isolates (meropenem minimum inhibitory concentration [MIC] ≥ 4 µg/mL and/or ertapenem MIC ≥ 2 µg/mL) (CLSI 2023) that were cataloged and stored by the Clinical Microbiology Laboratory at a New York City medical center over a 10-year period. Between 2009 and 2014, this collection consisted predominantly of bloodstream isolates, whereas between 2014 and 2018, cultures from the bloodstream and other nonbloodstream body sites were included. From this collection of CRE isolates, the first available isolate from each bacterial species per patient was included in the analysis. Multiple isolates were permitted for patients infected or colonized with more than one carbapenem-resistant bacterial species. All isolates underwent Illumina sequencing as previously described (Gomez-Simmonds et al. 2018b). Briefly, genomic DNA was extracted from bacterial broth cultures using the QIAamp DNA Blood Mini Kit or QIAamp 96 QIAcube HT DNA isolation Kit (Qiagen). Index-tagged whole-genome libraries were prepared using the Illumina DNA Prep workflow and sequenced on a MiSeq system (Illumina). We then screened CRE isolates for the presence of *bla*_KPC_ using SRST2 (Inouye et al. 2014) to map short-read data to the ARG-ANNOT v3 (Gupta et al. 2014) reference database. In addition, short reads were mapped against the PubMLST (Jolley et al. 2018) and PlasmidFinder (Carattoli et al. 2014) databases to determine the isolate MLST and plasmid profile of each isolate, respectively, and TETyper (Sheppard et al. 2018) was used to identify *Tn*4401 variants associated with *bla*_KPC_. Long-read nanopore sequencing, hybrid assembly, and plasmid analysis were performed on isolates harboring *bla*_KPC_ as described below. Study procedures were approved by the Columbia University IRB (protocol #AAAP3617 and AAAR7701).

### Nanopore sequencing and hybrid assembly

Our nanopore sequencing approach has been described previously (Gomez-Simmonds et al. 2018b, 2022). For all isolates in which *bla*_KPC_ was detected (*n* = 460), the Rapid Barcoding Kit (SQK-RBK004 or RBK110.96) was used to prepare multiplexed DNA libraries for nanopore sequencing on R9.4.1 flow cells using the MinION or GridION (Oxford Nanopore Technologies). Basecalling and demultiplexing were performed by MinKNOW (Oxford Nanopore Technologies); reads were then trimmed using Porechop v0.2.1 (Wick et al. 2017a). We generated hybrid assemblies from Illumina and nanopore reads using Unicycler v0.4.8 (Wick et al. 2017b). Resulting hybrid assembly graphs were visually reviewed using Bandage (Wick et al. 2015), including applying integrated BLAST searches (Camacho et al. 2009) to identify nodes coharboring plasmid replicon genes and *bla*_KPC_. Isolates were resequenced as necessary to produce assemblies with closed, circular *bla*_KPC_-harboring plasmid sequences without ambiguities. We were unable to generate complete plasmid assemblies for 25/460 isolates despite resequencing, which we hypothesized could be due to plasmid loss and/or the presence of mixed populations of recombinant plasmids in frozen stock cultures, and excluded these isolates from the final analysis. Predicted coding regions were annotated in final assemblies using Prokka v1.14.6 (Seemann 2014). Kleborate v2 (Lam et al. 2021) and hsp60ECCtool (https://github.com/karubiotools/hsp60ECCtool) were used to delineate species identity within *K. pneumoniae* and *E. cloacae* complexes, respectively. For comparison, for a subset of isolates, we generated assemblies using Illumina short-read data only SPAdes (Prjibelski et al. 2020).

### Phylogenetic analysis

Within each ST comprising at least two isolates, we assessed pairwise genetic differences to identify closely related isolates likely belonging to the same transmission network. A complete chromosomal assembly was selected from an isolate within each ST to serve as a reference genome; we chose the isolate from the midpoint of the collection to maximize core genome size and did not exclude prophage or recombination regions, as suggested by Gorrie et al. (2021), particularly given the concern that recombination masking could inappropriately diminish genetic distances in isolates with larger areas of recombination. Core genome SNPs were identified by mapping other isolate genomes to the reference genome using Snippy v3 (https://github.com/tseemann/snippy). Organisms lacking an established ST scheme were grouped by species. A threshold of ≤25 SNPs was used to define pairs of closely related isolates (Gorrie et al. 2021; Sherry et al. 2021); all isolates belonging to a pair differing by fewer SNPs than the defined SNP threshold were designated as belonging to the same clonal transmission cluster or “clade.” Histograms of pairwise SNP distances were also used to assess the appropriateness of this threshold for different STs (see Supplemental Fig. S4). ST258 isolates were also subdivided by their *cps* locus genotype based on *wzi* typing (Brisse et al. 2013; Wyres et al. 2015). Phylogenetic trees were constructed for *K. pneumoniae* ST258 and *E. cloacae* ST171 isolates based on core genome concatenated SNPs using RAxML v8 with GTR-GAMMA-based likelihood estimation (Stamatakis 2014). Support for nodes was assessed using 100 rapid bootstrap inferences, and a final tree was selected through a maximum likelihood search under Gamma. Phylogenetic trees were visualized using iTOL v6 (Letunic and Bork 2007). Clear outgroups within each ST were used to root each phylogenetic tree.

### Plasmid clustering and comparative sequence analysis

To characterize differences across *bla*_KPC_-harboring plasmid genomes, plasmid sequences were extracted and underwent comprehensive typing using the MOB-typer tool from the MOB-suite (Robertson and Nash 2018), which provides replicon and relaxase typing as well as mobility prediction (e.g., whether the plasmid is conjugative or mobilizable). We also used MOB-cluster to create a closed plasmid reference database for identifying closely related plasmids within our data set based on Mash distance thresholds. Here, we defined plasmid clusters as groups of plasmids belonging to the same secondary cluster (Mash distance threshold 0.025), which was previously shown to optimize plasmid cluster detection (Garcillán-Barcia et al. 2023). Plasmids that did not share a primary (Mash distance 0.06) or secondary MOB-cluster designation with any other plasmids in the collection were termed sporadic. Sequences belonging to the same primary plasmid cluster were also aligned and manually compared using progressiveMauve (Darling et al. 2010) in Geneious Prime (Biomatters) to review the overall structure and shared contents. Finally, plasmids from our collection were compared against plasmids from the NCBI Nucleotide database (https://www.ncbi.nlm.nih.gov/nucleotide/) that were collected in PLSDB to identify closely related plasmids with national and global distribution using the Mash distance algorithm with a maximum distance of 0.06 (Galata et al. 2019). We then used BLAST Ring Image Generator (BRIG) to visualize comparisons between closely related plasmids identified in PLSDB and study plasmids (Alikhan et al. 2011). We also performed a second plasmid clustering analysis including plasmids identified in CRE isolates from a cohort of liver transplant recipients undergoing prospective, longitudinal surveillance for intestinal colonization with CRE and other multidrug-resistant bacteria (Supplemental Table S2). Our approach to patient enrollment, serial collection of fecal samples from study participants, and stool culture for isolation of CRE have been described previously (Macesic et al. 2018; Annavajhala et al. 2019). We performed DNA extraction and nanopore long-read sequencing on these CRE isolates as described above; isolates harboring *bla*_KPC_ underwent whole-genome assembly using Flye v2.9.3 (Kolmogorov et al. 2019). We then extracted plasmid sequences for inclusion in a database with plasmids from our clinical CRE isolates for analysis using MOB-cluster.

### Incidence of horizontal transfer

All isolates belonging to the same clade within a given ST were designated as having undergone clonal transmission regardless of their plasmid contents (Fig. 1). Isolates that were not included in a clonal transmission cluster only were categorized as having acquired *bla*_KPC_ by plasmid transmission if they harbored a plasmid found in at least one other unrelated isolate (i.e., an isolate that did not belong to the same clade or ST). For isolates with multiple *bla*_KPC_-harboring plasmids, plasmid transmission was adjudicated based on having at least one shared plasmid with another isolate. All other isolates were deemed to have an unknown transmission mechanism. To estimate the incidence proportion of horizontal transfer at our hospital during the study period we calculated the following:

$$\begin{array}{rl} & \text{\% }\mathrm{horizontal}\;\mathrm{transfer}=\mathrm{plasmid}\;\mathrm{transmission}/\\ & \;(\mathrm{plasmid}\;\mathrm{transmission}+\mathrm{clonal}\;\mathrm{transmission}). \end{array}$$

Here isolates with an unknown transmission mechanism (i.e., that did not meet the criteria for clonal or plasmid transmission) were excluded. In addition, we repeated this calculation including only isolates collected between 2014 and 2018, as our isolate collection was more complete during this period and thus thought to more closely approximate an epidemiologic data set.

## Data access

CRE genomes generated in this study have been submitted to the NCBI BioProject database (https://www.ncbi.nlm.nih.gov/bioproject/) under accession numbers PRJNA1088550 and PRJNA759273. Short- and long-read data generated in this study have been submitted to the NCBI BioProject database under accession number PRJNA1133668 (see Supplemental Table S1 for isolate accession numbers). Assemblies including plasmid sequences from liver transplant recipients generated in this study have been submitted to the NCBI BioProject database under accession number PRJNA1144492 (see Supplemental Table S2).

## Supplemental Material

Supplement 1

Supplement 2

Supplement 3
