# Supplementary material for: Genomic epidemiology of carbapenem-resistant Enterobacterales at a New York City hospital over a 10-year period reveals complex plasmid-clone dynamics and evidence for frequent horizontal transfer of blaKPC
Source: Genome Res. 2024 Nov;34(11):1895–907. doi: 10.1101/gr.279355.124 (PMC11610580; doi:10.1101/gr.279355.124)
Supplement: Supplement 1 [file Supplemental_Material.pdf]

Supplemental Material

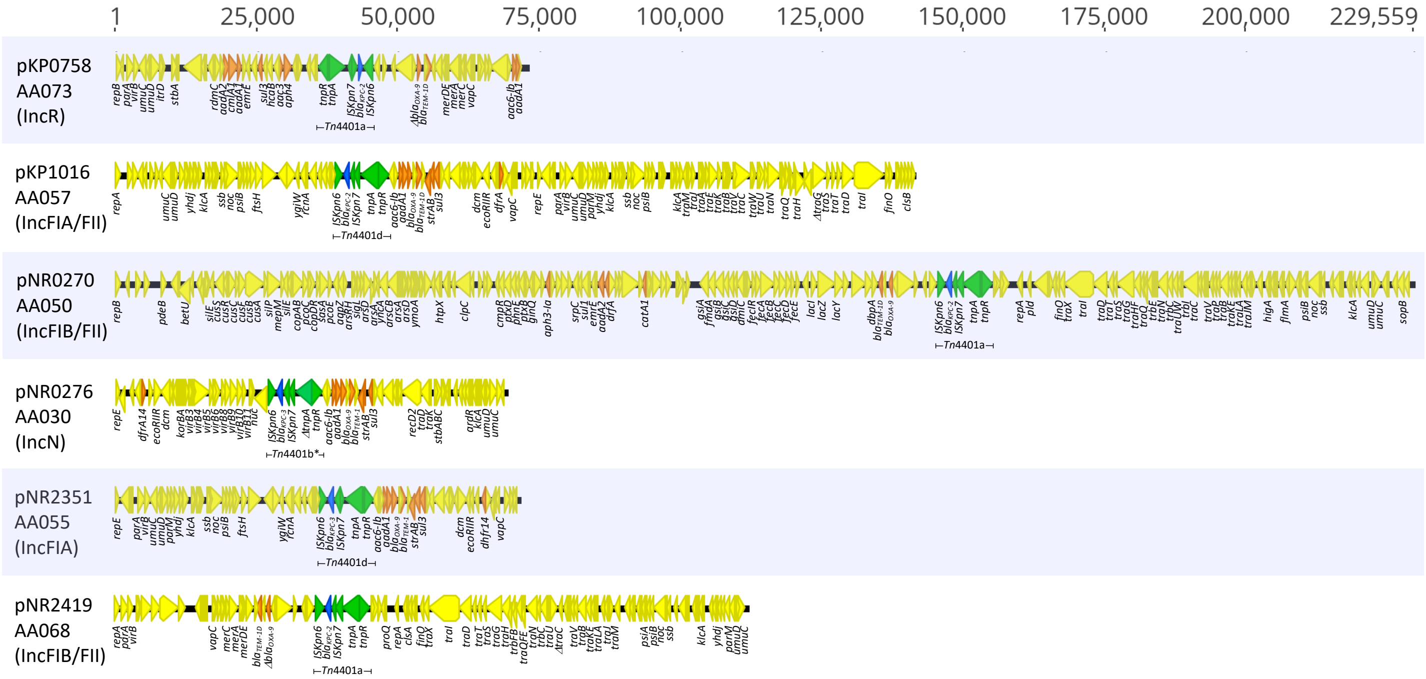

Supplemental Figure S1. Genomic structure of representative plasmids from each of the most common *bla*$_{KPC}$-harboring plasmid clusters found in this collection of clinical carbapenem-resistant Enterobacterales (CRE) isolates. *bla*$_{KPC}$ is shown in blue, other genes found within the Tn*4401* transposon are shown in green, other antibiotic resistance genes are in orange, and all other predicted coding sequences are shown in yellow.

A

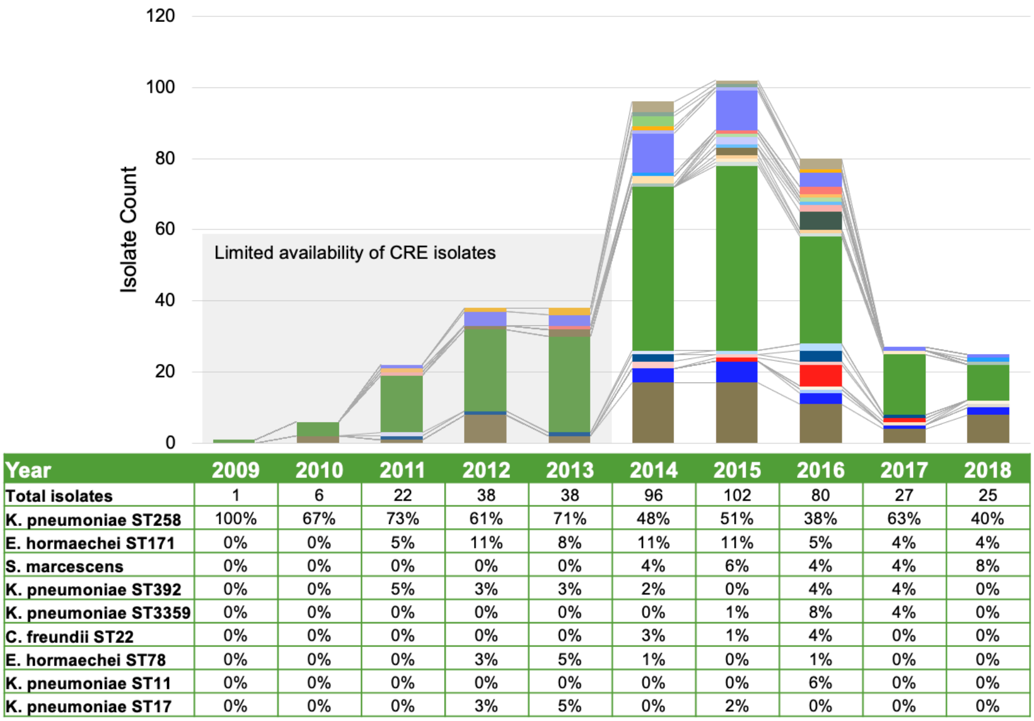

Year	2009	2010	2011	2012	2013	2014	2015	2016	2017	2018
Total isolates	1	6	22	38	38	96	102	80	27	25
K. pneumoniae ST258	100%	67%	73%	61%	71%	48%	51%	38%	63%	40%
E. hormaechei ST171	0%	0%	5%	11%	8%	11%	11%	5%	4%	4%
S. marcescens	0%	0%	0%	0%	0%	4%	6%	4%	4%	8%
K. pneumoniae ST392	0%	0%	5%	3%	3%	2%	0%	4%	4%	0%
K. pneumoniae ST3359	0%	0%	0%	0%	0%	0%	1%	8%	4%	0%
C. freundii ST22	0%	0%	0%	0%	0%	3%	1%	4%	0%	0%
E. hormaechei ST78	0%	0%	0%	3%	5%	1%	0%	1%	0%	0%
K. pneumoniae ST11	0%	0%	0%	0%	0%	0%	0%	6%	0%	0%
K. pneumoniae ST17	0%	0%	0%	3%	5%	0%	2%	0%	0%	0%

B

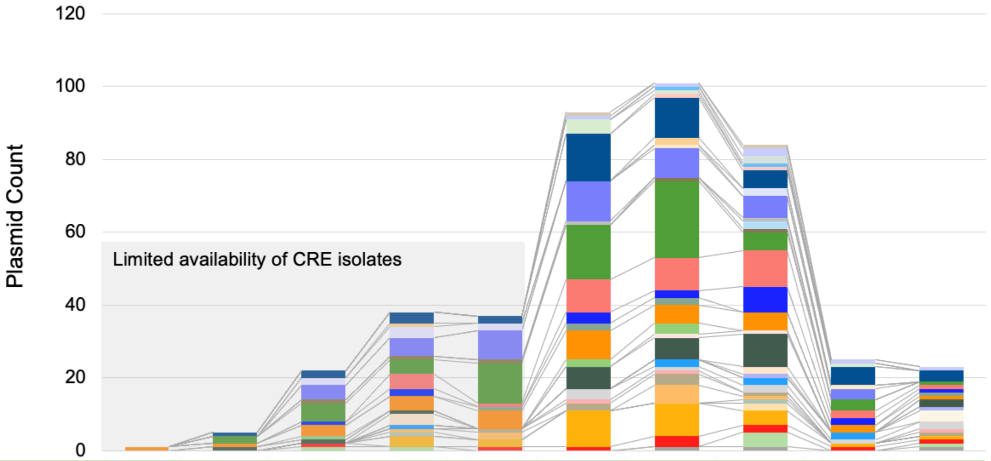

Year	2009	2010	2011	2012	2013	2014	2015	2016	2017	2018
Total CRE isolates	1	6	22	38	38	96	102	80	27	25
Total plasmids	1	5	22	38	37	93	101	84	25	23
AA029 (IncA/C)	0.0%	0.0%	4.5%	0.0%	2.7%	1.1%	3.0%	2.4%	4.0%	4.3%
AA030 (IncN)	0.0%	0.0%	0.0%	7.9%	5.4%	10.8%	8.9%	4.8%	4.0%	4.3%
AA046 (Nontyped)	0.0%	20.0%	4.5%	2.6%	0.0%	6.5%	5.9%	10.7%	0.0%	8.7%
AA050 (IncFIB/FII)	100.0%	20.0%	13.6%	10.5%	13.5%	8.6%	5.0%	6.0%	8.0%	4.3%
AA053 (IncFIA/FII)	0.0%	0.0%	4.5%	5.3%	0.0%	3.2%	2.0%	8.3%	8.0%	4.3%
AA055 (IncFIA)	0.0%	0.0%	0.0%	10.5%	2.7%	9.7%	8.9%	11.9%	8.0%	4.3%
AA057 (IncFIA/FII)	0.0%	40.0%	22.7%	10.5%	29.7%	16.1%	20.8%	6.0%	12.0%	4.3%
AA068 (IncFIB/FII)	0.0%	0.0%	18.2%	13.2%	21.6%	11.8%	7.9%	7.1%	12.0%	0.0%
AA073 (IncR)	0.0%	20.0%	9.1%	7.9%	5.4%	14.0%	10.9%	6.0%	20.0%	13.0%

Supplemental Figure S2. Distribution of A) sequence types (STs) and B) plasmids (using plasmid cluster designations) across the ten year study period. The most common A) STs (≥5 isolates per ST) and B) plasmids (≥10 plasmids per cluster) are indicated in bold type in each corresponding legend, and yearly proportions shown beneath the graphs. Of note, CRE isolate availability was largely limited to bloodstream isolates prior to 2014.

Whole genome sequencing and analysis workflow

1. Illumina sequencing for comprehensive genotyping including determining isolate multilocus sequence type (ST)

2. Phylogenetic analysis to identify clades (groups of isolates with genetic distances ≤25 SNPs) within each ST

3. Nanopore sequencing and hybrid assembly to obtain complete plasmid sequences

4. MOB-cluster to identify plasmid clusters (plasmids belonging to the same secondary MOB-cluster)

Incidence calculations based on clade and plasmid designations

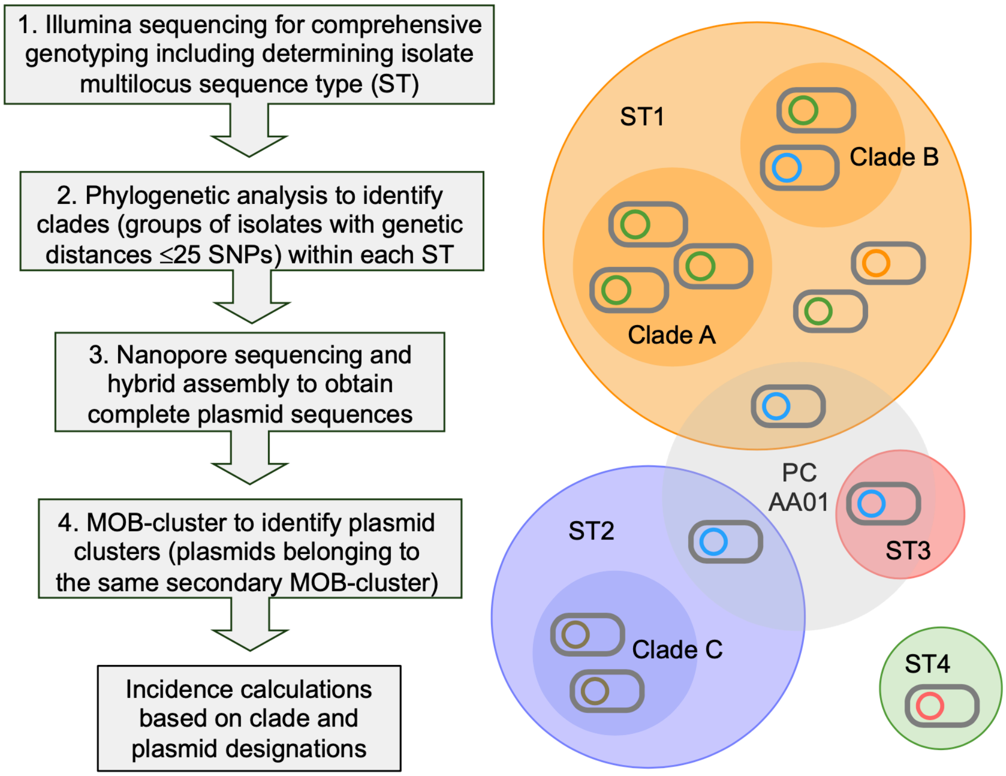

Cluster definitions

Clonal transmission: isolates belonging to the same ST and clade regardless of plasmid contents

Plasmid transmission: unrelated isolates harboring plasmids from the same plasmid cluster (PC)

Unknown transmission mechanism: isolates with a unique clonal background and plasmid

Supplemental Figure S3. Schematic of our whole genome sequencing and analysis workflow and cluster definitions.

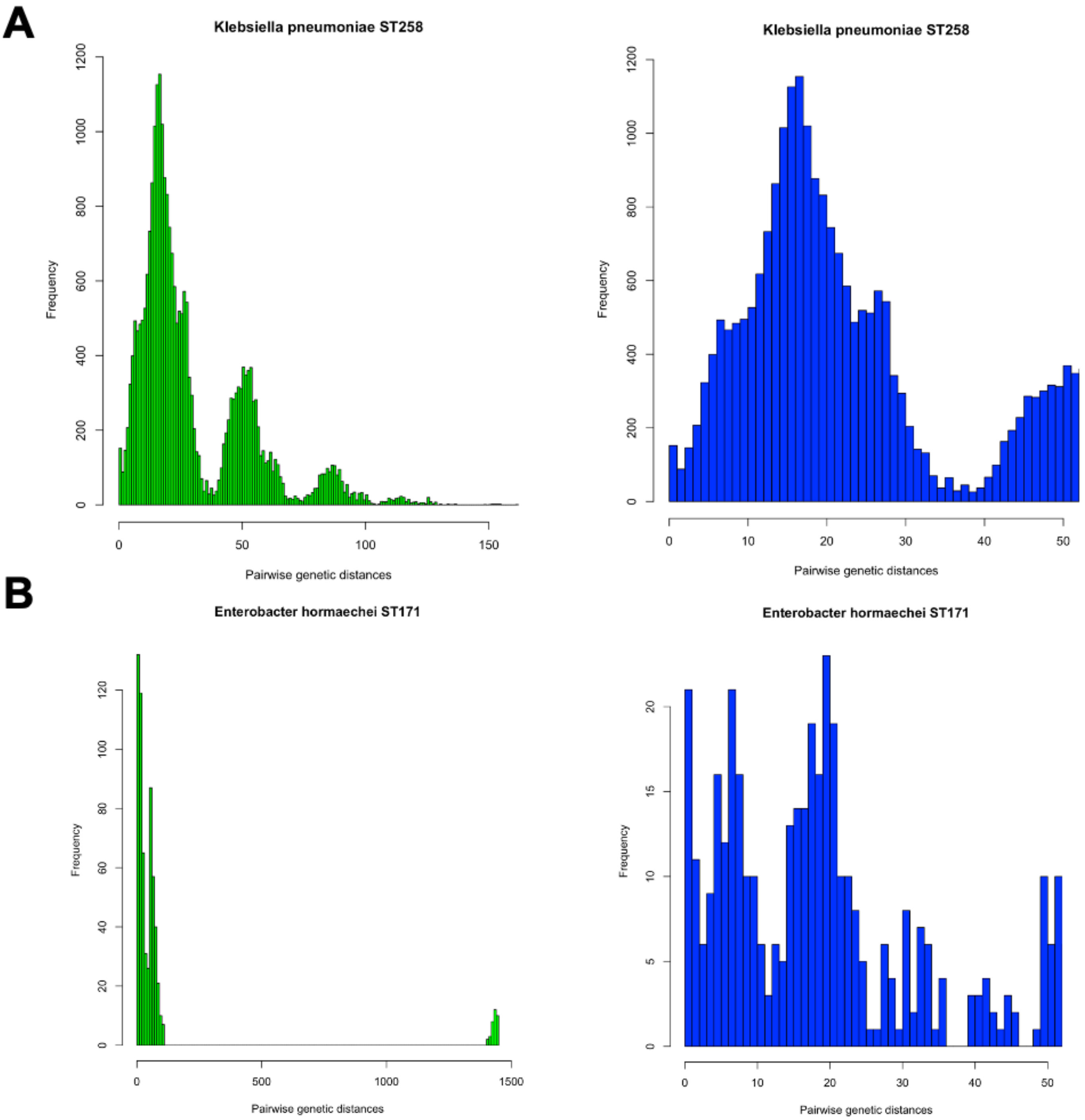

Supplemental Figure S4. Distribution of within-ST pairwise genetic distances for A) *Klebsiella pneumoniae* ST258 and B) *Enterobacter hormaechei* ST171. All genetic distances are included on the left (green), whereas maximum SNP distance maximum values are limited to 50 on the right (blue).
